# Encapsulation of ovarian allograft precludes immune rejection and promotes restoration of endocrine function in immune-competent ovariectomized mice

**DOI:** 10.1038/s41598-019-53075-8

**Published:** 2019-11-12

**Authors:** James Ronald Day, Anu David, Mayara Garcia de Mattos Barbosa, Margaret Ann Brunette, Marilia Cascalho, Ariella Shikanov

**Affiliations:** 1 Department of Biomedical Engineering, University of Michigan, Ann Arbor, USA; 2 Department of Macromolecular Science & Engineering, University of Michigan, Ann Arbor, USA; 3 Department of Obstetrics and Gynecology, University of Michigan, Ann Arbor, USA; 4 Department of Surgery, University of Michigan, Ann Arbor, USA; 5 Department of Microbiology & Immunology, University of Michigan, Ann Arbor, USA

**Keywords:** Immunology, Endocrine reproductive disorders, Biomedical engineering

## Abstract

Premature ovarian insufficiency (POI) is a significant complication of cytotoxic treatments due to extreme ovarian sensitivity to chemotherapy and radiation. POI is particularly devastating for young girls reaching puberty, because it irreversibly affects their physical and cognitive development. Changes occurring during puberty determine their height, bone health, insulin responsiveness, lipid metabolism, cardiovascular health and cognition. The only available treatment for POI during puberty is hormone replacement therapy (HRT), which delivers non-physiological levels of estrogen, lacks other ovarian hormones and pulsatility, and is not responsive to feedback regulation. Here we report that ovarian allografts encapsulated in a hydrogel-based capsule and implanted in ovariectomized mice restore ovarian endocrine function in immune competent mice. Ovarian tissue from BALB/c mice was encapsulated in poly(ethylene-glycol) (PEG) hydrogels, with a proteolytically degradable core and a non-degradable shell. The dual capsules were implanted subcutaneously in immune competent ovariectomized C57BL/6 mice for a period of 60 days. As expected, non-encapsulated ovarian allografts implanted in a control group sensitized the recipients as confirmed with donor-specific IgG in the serum, which increased 26-fold in the 3 weeks following transplantation (p = 0.02) and infiltration of the graft with CD8 T cells consistent with allo-immunity. In contrast, encapsulation in the Dual PEG capsules prevented sensitization to the allograft in all the recipients with no evidence of lymphocytic infiltration. In summary, the approach of hydrogel-based immunoisolation presents a minimally invasive and robust cell-therapy to restore hormonal balance in ovarian insufficiency. This report is the first to demonstrate the application of a tunable PEG-based hydrogel as an immunoisolator of allogeneic ovarian tissue to restore endocrine function in ovariectomized mice and prevent cell-mediated immune rejection in immune competent mice.

## Introduction

## Ovarian Endocrine Function Restoration for Cancer Survivors with Premature Ovarian Failure

Childhood cancer survival rates have significantly increased reaching over 80% in 2017 due to improved cancer therapies; however, the same life-saving anticancer treatments have led to profound health complications in the young survivors^1–6^. One of the most significant side effects of the anticancer therapy is ovarian toxicity, leading to impaired gonadal function and premature ovarian insufficiency (POI)^7,8^. POI is particularly devastating for young girls reaching puberty, because it irreversibly affects their physical and cognitive development. Changes occurring during puberty promote the physical and psychologic development into adulthood determining height, bone health, insulin responsiveness, lipid metabolism, cardiovascular health and cognition^9–11^. These changes are initiated before puberty and orchestrated by the pulsatile secretion of gonadotropin releasing hormone (GnRH) and growth hormone (GH) from the hypothalamus, which in turn regulate the release of gonadotropins, luteinizing (LH) and follicle-stimulating (FSH) hormones from the pituitary. FSH and LH stimulate the ovaries to produce estradiol, androstenedione, progesterone, inhibins A and B, activin and follistatin which have systemic effects in many organs and tissues including endocrine glands and the regulation of reproductive functions.

Hormones secreted from the ovary create a negative feedback loop by inhibiting the production of GnRH and FSH in the brain, resulting in dynamic oscillating levels of circulating hormones and gonadotropins^12–14^. The pulsatility and the circadian rhythm of the hypothalamic-pituitary-gonadal (HPG) axis are crucial for the development and regulation of a multitude of systems including the reproductive, fat, musculoskeletal, cardiovascular, and immune systems^15,16^. Deficiency of gonadal hormones, as occurs in POI, leads to irreversible imbalance in bone development and metabolic abnormalities. Thus, POI impedes normal puberty causing stunted bone growth, abnormal fat distribution and deposition and metabolic changes^8,17,18^.

The only available treatment to initiate puberty in girls with POI is hormone replacement therapy (HRT). HRT was originally designed to treat postmenopausal symptoms in women and long-term safety data in children are scant^19^. For puberty induction HRT is usually initiated with low levels of estrogen, followed by increasing doses of estrogen and progesterone combination as a life-long treatment, until menopause^20,21^. Unfortunately, HRT delivers only a fraction of ovarian hormones, e.g. estrogen and progesterone, at higher doses and at a constant and non-pulsatile rate, which is inadequate to reproduce physiological puberty^22^. Achieving an optimal pharmaceutical delivery of all the ovarian hormones is challenging due to the complexity of the regulation of the HPG axis. As a result, HRT-mediated puberty in girls with POI results in premature closure of the bone growth plate, cessation of bone growth and long-term metabolic imbalances^2,5,23,24^.

On the other hand, cryopreservation of ovarian tissue prior to gonadotoxic treatments followed by autotransplantation is a promising novel experimental approach, which restores fertility and ovarian endocrine function. This practice has already resulted in more than 70 babies born to identical twin sisters and cancer survivors who cryopreserved their ovaries^25–30^. However, in patients with hematological malignancies, this procedure cannot be performed because of the risk of introducing malignant cells present in the ovarian tissue, nor would it be helpful for patients who did not cryopreserve their tissue before the anti-cancer treatments, which by far is the most common case^31–34^.

To mitigate the limitations of HRT and to avoid the risk of cancer recurrence associated with autotransplantation of ovarian tissue from subjects with hematologic cancers, we have developed a novel strategy to restore ovarian endocrine function. In this study we investigated whether a synthetic viscoelastic poly(ethylene glycol vinyl sulfone) (PEG-VS) hydrogel sustains survival and function of allogeneic ovarian tissue while preventing rejection. The capsule provides a supportive environment for the encapsulated and implanted tissue, allows diffusion of oxygen and nutrients, and physically prevents cell migration. Our earlier studies demonstrated that ovarian follicles encapsulated in proteolytically degradable PEG-VS hydrogels developed to the antral stages *in vitro*^35,36^ and *in vivo*^37^. By keeping the follicular structure intact we attempted to mimic the physiological interactions between the follicular cells and to maintain the follicle symphony^38–40^. Additionally, non-degradable PEG-VS matrices conducive for tissue encapsulation evoked minimal inflammatory response after implantation in a syngeneic mouse model^41,42^. The objective of this study was to investigate whether PEG-VS-based capsules support the survival and function of ovarian allografts and protect them from rejection, in absence of immune-suppression, in immune competent mice with POI.

## Materials and Methods

### Experimental design

**Figure 1:**
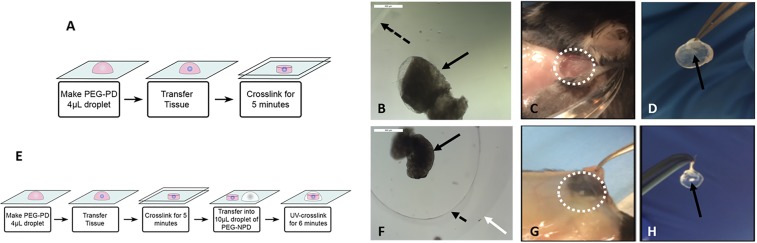
(**A**) Schematic of ovarian tissue encapsulation in PEG-PD. (**B**) Ovarian tissue encapsulated in PEG-PD before implantation, (**C**) at time of sacrifice, and (**D**) after removal. (**E**) Schematic of ovarian tissue encapsulation in Dual PEG. (**F**) Ovarian tissue encapsulated in Dual PEG before implantation, (**G**) at time of sacrifice, and (**H**) after removal. Black arrow indicates ovarian tissue, dotted black arrow points to PEG-PD core, white arrow points to the non-degradable PEG-based shell of the Dual PEG, and white dotted circle in C and G indicates area of gel. Drawings used to depict the encapsulation process were created using Adobe Illustrator (23.0.3 2019).

Adult cycling female mice (C57BL/6) underwent bilateral ovariectomies to induce POI. Ovaries from BALB/c 6–8 days old mice were encapsulated in (i) a proteolytically degradable PEG-VS hydrogel (PEG-PD) (Fig. 1A), (ii) a dual PEG-VS hydrogel (Dual PEG) containing a proteolytically degradable PEG-VS core with a non-degradable shell and subcutaneously implanted for 60 days (n = 10 recipient mice) (Fig. 1E) and (iii) TheraCyte, a commercially available poly-terephthalate-ethylene (PTFE) pouch. TheraCyte was used as a control for immune-protective capabilities as it has been used towards islet transplantation. All the encapsulated ovarian allografts were implanted subcutaneously for 60 days (n = 10 recipient mice). In the control group, non-encapsulated allogeneic ovaries from 6–8 days old BALB/c mice were subcutaneously implanted in C57BL/6 mice (n = 9) for 28 or 60 days. The number of donor mice matched the number of recipient mice with each recipient mouse receiving 2 BALB/c ovaries.

### Ovariectomies in recipient mice

The IACUC guidelines for survival surgery in rodents and the IACUC Policy on Analgesic Use in Animals Undergoing Surgery were followed for all the procedures. Animal experiments for this work were performed in accordance with the protocol approved by the Institutional Animal Care and Use Committee (IACUC) at the University of Michigan (PRO00007716).

Bilateral ovariectomies were performed on adult female mice (C57BL/6) aged 12–16 weeks. The mice were anesthetized by isoflurane. Carprofen (5 mg/kg. body weight, Rimadyl, Zoetis) was administered subcutaneously for analgesia. The intraperitoneal space was exposed through a midline incision in the abdominal wall secured using an abdominal retractor. The ovaries were removed, and the muscle and skin layer of the abdominal wall were closed with 5/0 absorbable sutures (AD Surgical). The mice recovered in a clean warmed cage and received another dose of Carprofen 12 hours post recovery or as needed.

### Collection of donor ovaries

Ovaries from 6 to 8 days old BALB/c mice were collected and transferred to Leibovitz L-15 media (Sigma-Aldrich, USA). The ovaries were dissected into 2–4 pieces and transferred in the maintenance media (α-MEM, Gibco, USA) and placed in the CO_2_ incubator for further manipulation.

### Peg-vs hydrogel preparation and ovarian tissue encapsulation

To prepare PEG-PD hydrogels, 8-arm PEG-VS (40 kDa, Jenkem Technology, Beijing, China) was cross-linked with a plasmin sensitive tri-functional peptide sequence (Ac-GCYK↓NSGCYK↓NSCG, MW 1525.69 g/mol, > 90% Purity, CelTek, ↓ indicates the cleavage site of the peptide). Dual PEG hydrogels were prepared using 4-arm PEG-VS 20 kDa, Jenkem Technology) with Irgacure 2959 (Ciba, Switzerland, MW = 224.3) and 0.1% N-vinyl-2-pyrrolidone (Sigma-Aldrich, St. Louis, USA). The detailed protocol is described in Day *et al*.^42^ Donor ovaries were collected from 6–8 days old BALB/c mice. The collected ovaries were transferred to Leibovitz L-15 media (Sigma-Aldrich, USA) and dissected open. The ovarian tissue was then transferred into maintenance media (α-MEM; Gibco, USA), kept at 37 °C and 5% CO_2_. For encapsulation in the PEG-PD, the ovarian tissue was transferred into a 10 μL droplet of the plasmin sensitive tri-functional peptide and PEG-VS precursors’ solution. The droplet was allowed to crosslink for 5 minutes and then was quenched in maintenance media (Fig. 1A). For Dual PEG hydrogel encapsulation, the tissue was first encapsulated in a 4 μL PEG-PD hydrogel and was then placed in the center of a 10 μL bead of PEG-VS precursor solution (5% w/v PEG-VS, .4% Irgacure 2959, 0.1% NVP) and exposed to UV light for 6 minutes(Fig. 1E). All constructs were imaged immediately after encapsulation of the tissue (Fig. 1B,F).

### Ovary encapsulation in theracyte

The port of the TheraCyte was widened using a Hamilton syringe without compromising the integrity of the TheraCyte wall. The ovarian fragments were aspirated into a pipette and inserted into the TheraCyte. To verify whether all the ovarian pieces were in the TheraCyte, the transparent plastic port and the pipette were inspected under the stereo microscope. The long port tube was shortened and sealed by melting the plastic to prevent leakage of the ovarian pieces and invasion of the host cells. Each TheraCyte contained ovarian tissue from two ovaries, and each recipient (C57BL/6) was implanted with one TheraCyte.

### Subcutaneous implantation

A small incision was made on the dorsal side of the anesthetized mice (C57BL/6) and the immunoisolators (PEG-PD, Dual PEG and TheraCyte) with the ovarian tissue were implanted subcutaneously. The skin was closed using 5/0 absorbable sutures. The mice received Carprofen for analgesia for at least 24 hours after surgery or as needed. Mice were monitored for two months and euthanized at the end of the experiment. Control mice received empty PEG-VS hydrogels or non-encapsulated ovarian tissue (BALB/c, without a capsule) that were implanted subcutaneously for 28 days.

### Serum hormone analysis

Every two weeks blood was collected from the lateral tail vein at set time points, up to 1% of the total volume of the blood with a 5 ^**3/4**^” glass Pasteur pipette. At the time of sacrifice, blood was collected via cardiac puncture. Following blood collection, samples were stored at 4 °C overnight, then centrifuged for 10 minutes at 10,000 rpm and the separated serum was stored at −20 °C. The samples were analyzed for mouse FSH using a radio-immunoassay (Ligand Assay and Analysis Core Facility, University of Virginia Center for Research in Reproduction).

### Vaginal cytology

Daily vaginal cytology was used to establish presence of estrous cycle following ovariectomies and restoration of ovarian endocrine function after implantation of ovarian allografts. The loss of estrous cycle as a result of ovariectomies was confirmed by a persistence of leukocytes, which also correlated with elevated levels of circulating follicle stimulating hormone (FSH). The cyclical transition of cells from leukocytes to cornified and then to nucleated cells signaled resumption of normal estrous cyclicity and restoration of ovarian endocrine function.

### Histological analysis of the retrieved devices and the encapsulated ovarian tissue

Following sacrifice, the immunoisolating devices were retrieved from mice, fixed in Bouin’s fixative at 4 °C overnight, transferred and stored in 70% ethanol at 4 °C. After processing, samples were embedded in paraffin, serially sectioned at 5 μm thickness, and stained with hematoxylin and eosin.

### Flow cytometry

Serum allo-antibody titer measurements were performed using flow cytometry before and after implantation and reported as mean fluorescence intensities (MFI) for the highest dilution showing fluorescence detectable above background (non-immune serum from a non-implanted mouse) in immunized mice (positive controls implanted with allogeneic ovaries without a device). Thymocytes were isolated from BALB/c mice and incubated with serially diluted recipient serum for 30 minutes at 4 °C. Antibodies bound to the thymocytes were detected by Cy5-conjugated goat anti-mouse IgG (1:250 dilution, 1030–15, Southern Biotech) and Alexa Fluor 488-conjugated goat anti-mouse IgM (1:250 dilution, 1020–30, Southern Biotech) for 30 minutes at 4 °C and analyzed in a BD FACSCanto II (BD Biosciences, Franklin Lakes, NJ). The mean fluorescence intensities (MFI) in the APC-channel (measuring bound IgG) and FITC channel (measuring bound IgM) were determined with FlowJo 10 software (FlowJo, LLC, Ashland, OR).

### Immunohistochemistry for T cells

To analyze T cell infiltration following subcutaneous implantation with PEG-PD, Dual PEG, and TheraCyte, paraffin-sectioned slides were stained to identify CD4 and CD8+ cells. First, sections were deparaffinized with Xylene and rehydrated. The slides were incubated in antigen retrieval buffer, pH9.0 (ab94681, Abcam) for 20 minutes at 97 °C and additional 20 minutes at room temperature to cool down. Next, the slides were incubated with KPL Universal Block (5560–0009, Sera-Care) to block non-specific binding sites for 30 minutes at room temperature. The sections were incubated at room temperature for 1 hour with primary antibodies: rabbit monoclonal anti-mouse CD4 antibody (1:1000 dilution, ab183685, Abcam), rabbit polyclonal anti-mouse CD8 antibody (1:500 dilution, ab203035, Abcam). The slides were subsequently incubated at room temperature with secondary antibodies: goat anti-rabbit Ig (1:100 dilution for 15 minutes, 4010–05, Southern Biotech) for CD4 and goat anti-rabbit (1:50 dilution for 30 minutes, 4010–05, Southern Biotech) for CD8. Diaminobenzidine (BDB2004L, Betazoid DAB Chromogen kit, BioCare Medical) was used as a chromogen for 10 minutes at room temperature. Hematoxylin (220–102, Fischer Scientific) was used as a counterstain. For negative-controls, paraffin sections were incubated without the primary antibody. To assess presence or absence of CD8+ cells, 12 sections from the front, middle, and end of each specimen were examined, to represent the full thickness of the implant.

### Statistics

Statistical analysis was performed using the R software, version 3.1.2 (2014). Significance was determined by Welch’s two-sample t-test. To determine whether there was a significant difference between the ability of capsule to prevent rejection or not compared to controls, Pearson’s Chi-squared test was used. The two main outcomes of the test were 1) the functionality of the tissue and 2) the ability of the capsule to minimize the exposure of the allograft to the recipient’s immune system and minimize infiltration of the immune cells into the capsule. 1) Consistent estrous cyclicity and decrease in circulating FSH levels qualified as “functional grafts”, while absence of cycles and/or FSH levels similar to levels in ovariectomized deemed as “non-functional”. 2) Inability to detect circulating allo-antibodies and absence of CD8+ T cells in the allografts were deemed the capsules “immunoisolating”. Mice that presented with elevated levels of FSH, yet had undetectable levels of circulating allo-antibodies were classified as having a primary failure of the ovarian tissue for non-immunological reasons.

## Results

### Macroscopic evaluation of the ovarian implants

To determine physical integrity, all the encapsulating devices were macroscopically inspected at the time of retrieval after 60-day implantation period. The PEG-VS hydrogel-based capsules were easily identified and removed from the subcutaneous space. The ovarian tissue was visible at the center of the hydrogels. The PEG-based hydrogel capsules with ovarian allografts demonstrated minimal interaction with the host tissues at the implantation site (Fig. 1C,D,G,H). Similar to our findings from the syngeneic implantation^42^, macroscopically the capsules appeared intact and transparent, with no or minimal tissue attached and with no visible blood vessels or capillaries growing around or in the capsule.

**Figure 2:**
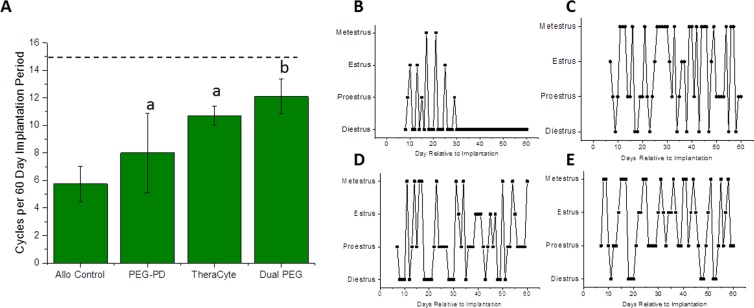
Restoration of estrous cycle in mice receiving encapsulated ovarian tissue. (**A**) Average number of cycles over the 60-day implantation period in mice receiving encapsulated ovarian tissue in PEG-PD, TheraCyte, and Dual PEG. Dashed line indicates the expected average number of cycles over 60 days, and “a” and “b” indicate significant differences, p < 0.05. Representative cycles of mice receiving (**B**) non-encapsulated ovarian tissue, (**C**) ovarian tissue encapsulated in PEG-PD, (**D**) ovarian tissue encapsulated in TheraCyte, and (**E**) ovarian tissue encapsulated in Dual PEG.

In contrast, the TheraCyte device, although intact, was firmly embedded in the subcutaneous space of the host. Multiple visible blood vessels surrounded the TheraCyte capsule. These findings are consistent with known properties of the PTFE surface promoting protein adsorption, interactions with cells and inflammatory changes in the surrounding tissues^43–46^. The controls ovarian allografts implanted without a device lasted less than 3 weeks after implantation and were completely destroyed leaving only intense vascularization due perhaps to immunity and inflammation (Supplemental Fig. 1).

### Restoration of ovarian endocrine function

We evaluated restoration of ovarian endocrine function by measuring the regularity of estrous cycles and circulating levels of FSH. In mice of reproductive age (starting at 4 weeks old) regular estrous cyclicity corresponds with normal ovarian function and ovulation. Each estrous cycle lasts 4–5 days and is easily identified by the type of cells present in the vaginal cytology sample. The estrous cycle corresponds to four stages: metestrus, estrus, proestrus and diestrus. After bilateral ovariectomy mice experience persistent diestrus and consistent with absence of ovarian function we found no cornified cells in the samples of vaginal cytology for a 2-week period.

After implantation of allogeneic ovarian tissue encapsulated in PEG-PD, TheraCyte, and Dual PEG mice demonstrated an average of 8, 11, and 12 cycles, respectively, over the 60-day implantation period (Fig. 2). All the mice (n = 9/9) implanted with non-encapsulated ovarian allograft (control group), resumed cyclicity one week after implantation. However, four weeks after the implantation all the control mice stopped cycling and returned to the continuous diestrus phenotype characteristic of POI, averaging at 5.75 cycles over a 60-day implantation period (Fig. 2A,B). In the PEG-PD group 90% of mice resumed cyclicity in the first week, however the rate of cyclicity continuously decreased and at the end of the experiment (60 days), only 50% of the mice were still cycling. Implantation of ovarian allograft encapsulated in TheraCyte restored cyclicity in 80% of the mice by the first week of implantation. The percentage of cycling mice steadily increased and and reached 100% after 4 weeks and remained steady up to day 60. Mice that received ovarian allograft encapsulated in Dual PEG experienced a delay in resumption of cyclicity compared to other mice, with only 50% of the mice cycling in the first week of implantation, 80% by the second and 100% by week 4. By the end of the implantation period, 80% of the mice receiving the Dual PEG implants were still cycling (Fig. 2B,C,D,E). Mice receiving allogeneic tissue encapsulated in Dual PEG exhibited the highest degree of estrous cycle resumption indicated by an average of 12 cycles over the 60 day implantation period (Fig. 2A), which is the expected amount of cycles for a non-menopausal mouse over that time span given the observed delay to resume estrous activity.

**Figure 3:**
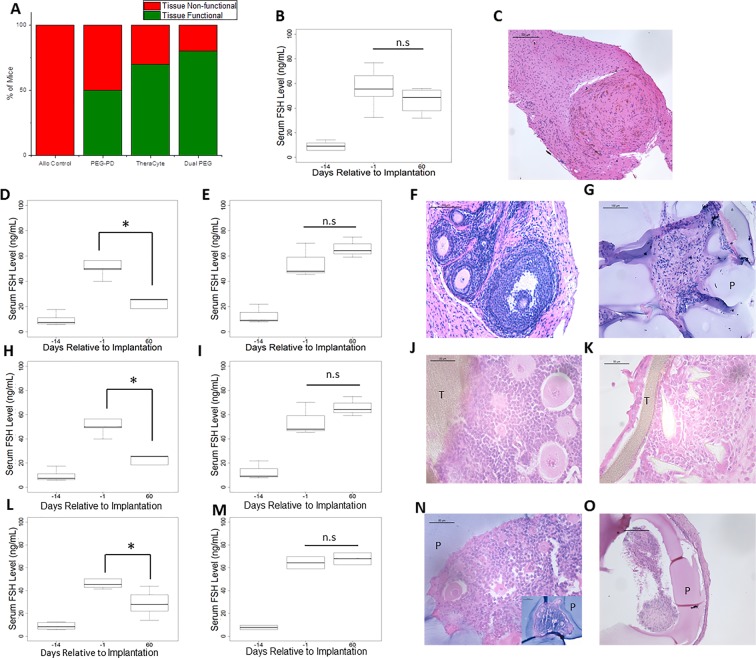
(**A**) Percentage of non-encapsulated ovarian tissue and encapsulated ovarian tissue in PEG-PD, TheraCyte, and Dual PEG that remained functional throughout the time course of the implantation or became non-functional. Consistent estrous cyclicity and decrease in circulating FSH levels qualified as “functional grafts”, while absence of cycles and/or FSH levels similar to levels in ovariectomized deemed as “non-functional”. (**B**) FSH levels of mice receiving non-encapsulated allogeneic ovarian tissue. (**C**) Histological image of non-encapsulated allogeneic ovarian tissue after 28 days post implantation. Serum FSH levels of (**D**,**H**,**L**) encapsulated ovarian tissue that remained functional or (**E**,**I**,**M**) became non-functional during the time course of the implantation in PEG-PD, TheraCyte, and Dual PEG, respectively. *Indicates statistical significance, p < 0.05, and n.s. represents not significant. Histological images of “functional” (**F**,**J**,**N**) or (**G**,**K**,**O**) “non-functional” encapsulated ovarian allografts in PEG-PD, TheraCyte, and Dual PEG, respectively, and retrieved 60 days after implantation. ‘−14’ corresponds to one day before ovariectomy and 2 weeks prior to implantation. ‘−1’ corresponds to two weeks after ovariectomy and 1 day prior to implantation. (**N inset**) Ovarian allografts containing healthy ovarian follicles were completely encapsulated in PEG through 60 days implantation. (P) indicates PEG, and (T) represents TheraCyte. Scale bars: 500 μm(**F**), 200 μm (**C**,**O**), 100 μm (**G**, **N inset**), 50 μm (**J**,**K**,**N**).

Circulating FSH and estradiol levels in healthy mice are inversely proportional and fluctuate during the estrous cycle because estradiol inhibits the production and secretion of FSH via negative feedback loop. Circulating FSH levels, serve as reliable surrogates for ovarian endocrine function. Before ovariectomies, mice in all the groups had an average FSH level of 9 ng/mL (n = 9 for the controls, n = 10 for the TheraCyte, n = 10 for the PEG-PD and n = 10 for Dual PEG). Following ovariectomies, FSH levels rose significantly to an average of 53 ng/mL, confirming the disruption of the HPG axis in all these mice (Fig. 3). In the control group, where mice received the non-encapsulated allogeneic ovarian tissue, FSH levels remained elevated during the 60-day implantation period, confirming insufficient production and secretion of ovarian hormones (Fig. 3B). In the PEG-PD group, five out of ten mice exhibited a significant decrease of FSH levels from 53 ng/mL to 23 ng/mL (p = 0.003) by day 60 and were deemed the “functional group” (Fig. 3A,D). The remaining five mice had an average FSH level of 56 ng/mL at day 60 similar to the levels before implantation (54 ng/mL) indicating absence of ovarian endocrine function, e.g. the “non-functional group” (Fig. 3A,E). In the TheraCyte implant group, seven out of ten mice were “functional” and had an average FSH level of 34 ng/mL after implantation compared to 50 ng/mL before implantation, (p = 0.02) (Fig. 3A,H). In three mice with TheraCyte implants, however, FSH levels did not decrease and reached an average of 63 ng/mL at 60 days indicating tissue failure. (Fig. 3A,I). The Dual PEG group had the greatest number of mice demonstrating estrous cyclicity and decreased levels of FSH. Based on estrous activity, the encapsulated tissue in Dual PEG was functional in eight of ten mice after 60 days of implantation. In these eight mice, we observed a significant decrease of FSH levels from 48 ng/mL to 28 ng/mL (p = 0.002) (Fig. 3A,L). The remaining two mice with the Dual PEG implants that stopped cycling by day-60 had an average FSH level of 68 ng/mL at day 60 indicating tissue failure (Fig. 3A,M).

To evaluate the follicular development of the implanted ovarian tissue we analyzed tissue sections explanted at 60 days after implantation by histological analysis. In controls transplanted with non-encapsulated ovarian allografts we observed no ovarian follicles at the site of implantation, consistent with graft rejection (Fig. 3C). In contrast, we observed all stages of ovarian follicle development (*up to the antral stage*) in sections obtained from the functional PEG-PD, TheraCyte and Dual PEG encapsulated allografts (Fig. 3F,J,N) indicating that those devices support follicle development. Multiple antral follicles were not observed in any of the groups. As expected, there were no structurally healthy-looking ovarian follicles in sections from non-functional PEG-PD, TheraCyte and Dual PEG implants (Fig. 3G,K,O).

### Ovarian allograft immunity

**Figure 4:**
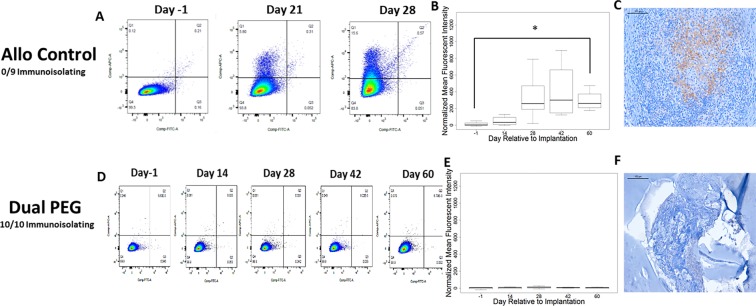
(**A**) Representative flow cytometry plots reflecting binding of serum allo-specific antibodies from recipients of non-encapsulated grafts, to donor cells. Y-axis, donor-specific IgG; X-Axis, donor-specific IgM. The mean fluorescence intensity (MFI) for donor specific IgG reflects the average of values read on the Y-Axis; the MFI for donor specific IgM reflects the average of values read on the X-Axis (**B**) Graph depicts the average MFI ± SD of allo-specific IgG in mice receiving non-encapsulated allogeneic ovarian tissue. *Indicates statistical significance (p < 0.05) (**C**) Immunohistochemical staining of CD8+ cells present in the non-encapsulated allograft. (**D**) Representative flow cytometry plots reflecting binding of serum allospecific antibodies, obtained from recipients of dual PEG-encapsulated grafts, to donor cells; and (**E**) Average MFI ± SD of allo-specific IgG in mice receiving allogeneic ovarian tissue encapsulated in Dual PEG. (**F**) Immunohistochemical staining of CD8+ cells in the dual PEG-encapsulated allograft. Scale bars: 100 μm(**C**), 200 μm(**F**). Inability to detect circulating allo antibodies and absence of CD8+ T cells in the allografts were deemed the capsules “immunoisolating”.

Efficacy of encapsulation to sustain ovarian allograft function depends on preventing host sensitization and/or destruction by allo-immunity effectors. Allo-specific immunity is manifested by the production of specific-antibodies and by infiltration of grafts by lymphocytes. To determine if encapsulated ovarian allografts evoked immunity we measured circulating allo-specific IgM and IgG by flow cytometry. Figure 4A,B demonstrate that donor-specific IgG increased 26 fold in the 3 weeks following transplantation (p = 0.02) of non-encapsulated ovarian allografts. As hypothesized, all of the ovarian allografts evoked allo-specific immunity (n = 9/9) (Fig. 4A,B). Immuno-pathology examination of the grafts revealed infiltration with CD8 T cells consistent with allo-immunity (Fig. 4C).

In contrast, encapsulation of the ovarian tissue in Dual PEG hydrogels blocked sensitization in all the recipient mice. None of the mice receiving ovarian allograft encapsulated in Dual PEG devices (n = 10/10 immunoisolating) produced allo-specific IgG up to 60 days after transplantation, at explant (Fig. 4D,E). Consistent with the absence of allo-immunity, immunopathology analysis of the ovarian allografts encapsulated in the Dual PEG device showed no evidence of lymphocytic infiltration. (Fig. 4F). These results indicate that the Dual PEG device has immune-isolating properties and suggests non-immunologic reasons, such as primary tissue-driven failure, for the absence of ovarian function in two Dual PEG device encapsulated allografts.

**Figure 5:**
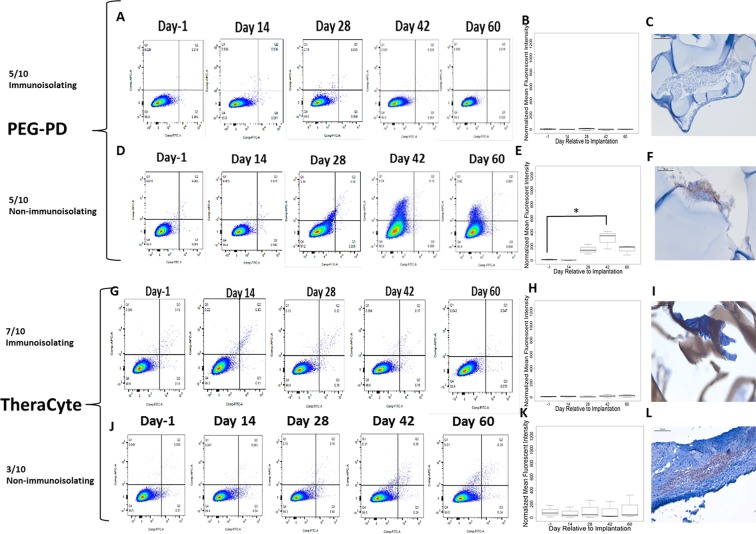
Representative flow cytometry plots reflecting binding of serum allo-specific antibodies from recipients of allogeneic ovary tissue encapsulated in PEG-PD or TheraCyte where the tissue (**A**,**G**) remained functional or (**D**,**J**) failed during the implantation period. Average MFI ± SD of allo-specific IgG levels in mice receiving PEG-PD and TheraCyte where the tissue (**B**,**H**) remained functional or (**E**,**K**) failed during the implantation period. Immunohistochemical staining of CD8+ cells present in mice receiving PEG-PD and TheraCyte where the tissue (**C**,**I**) remained functional or (**F**,**L**)failed. Scale bars: 100 μm (**C**,**F**,**I**,**L**).

Ovarian allografts encapsulated in degradable capsules, PEG-PD, and Theracyte produced mixed results in terms of ovarian function and allo-immunity (Fig. 5). Encapsulation in PEG-PD hydrogels was less effective at preventing sensitization than encapsulation with in Dual PEG hydrogels. Five out of 10 mice with allografts encapsulated in PEG-PD hydrogels had allo-specific IgG and CD8 T lymphocytes were detected in the excised graft. (Fig. 5D,E,F). In these mice, follicles were not observed and little viable tissue remained after 60 days, corroborating the tissue was not functional. Of the 10 mice implanted with ovarian allografts encapsulated in TheraCyte, three mice developed a an allo-specific IgM-IgG response and had CD8 lymphocytes in the graft (Fig. 5J,K,L). In summary we show that Dual PEG devices allow long-lived function of ovarian allographs while blocking sensitization of the host. Dual PEG devices were superior to TheraCyte or PEG-PD at maintaining function of the enclosed tissue (Fig. 3A).

## Discussion

In this study, we used PEG based hydrogel capsules to enclose and implant ovarian allografts in immune competent ovariectomized mice to restore the ovarian endocrine function in the hosts. We had demonstrated earlier that the proteolytically degradable synthetic PEG hydrogels (PEG-PD) cross-linked with protease sensitive peptides promote the survival and growth of encapsulated murine primordial ovarian follicles in a syngeneic mouse model of POI^37,42^. Our results show that encapsulation with PEG-PD and Dual PEG hydrogel sustain physiological development of ovarian allograft tissue *in vivo*. Dual PEG implants proved to be superior at maintaining function in the long run because they maintain structural integrity for longer that PEG-PD.

While the degradable PEG-PD hydrogels sustain ovarian follicle growth in a syngeneic model, we hypothesized that degradation of these hydrogels cannot support long-term survival and function of ovarian allografts because the immune cells from the host would eventually reach the allograft causing rejection. In fact, 60 days post implantation, 50% of PEG-PD ovarian allografts were “non-functional” similar to non-encapsulated allografts with elevated levels of circulating allo-specific IgG. We hypothesize the degradable matrix of PEG-PD resulted in larger pores over time, which allowed sensitization to occur and allowed host immune cells to infiltrate the hydrogel and cause further damage to the encapsulated tissue. Interestingly, only 50% of the hydrogels degraded enough to allow infiltration of the immune cells while the other 50% remained functioning with no detectable sensitization. These findings suggest that the kinetics of degradation of the PEG-PD hydrogels determines variable host responses and may introduce variability.

To combine the benefits of hydrogels at sustaining ovarian function and a more robust shell to avoid sensitization and early graft demise, we developed a Dual PEG hydrogel encapsulating system with a proteolytically degradable core to allow follicular development and a non-degradable outer shell to act as a barrier against the host immune system. In a syngeneic study, Dual PEG hydrogels implanted with ovarian tissue caused minimal inflammatory response and supported ovarian follicle survival and development, and promoted restoration of endocrine function in ovariectomized mice for at least 60 days^42^. In the current study, we investigated whether Dual PEG hydrogels protected the encapsulated ovarian allograft from the host immune system and restored ovarian endocrine function. The dual capsule was compared to TheraCyte, a commercially available immunoisolator experimentally used for allogeneic islet transplantation. Encapsulated islets have been shown to survive in TheraCyte in allogeneic models, however in human clinical trials, insulin independence was not achieved^47^. Recently, we showed that TheraCyte supports implantation of ovarian allograft for 21 days and protects the tissue from rejection^48^. We found the retrieved TheraCyte devices to be encapsulated and completely surrounded by the recipient tissue while the inert surface of the Dual PEG hydrogels resulted in minimal interactions with the host which is consistent with earlier findings^41,42^. The survival and function of ovarian allografts in Dual PEG was superior compared to allografts in the TheraCyte device. It is possible that the encapsulation and deposition of cells and extracellular matrix around the TheraCyte device contributed to decreased function. Additionally, the confined space in the rigid pouch of TheraCyte could restrict follicle expansion and further contributing to failure, while the modular design of the Dual PEG can accommodate follicle expansion and greater number of tissue fragments. Lastly, inclusion of the non-degradable shell in the Dual PEG capsules shielded the allogeneic ovarian tissue against an allogeneic response in contrast with what was observed with the PEG-PD alone.

Of those mice that did not experience rejection, a significant decrease in the levels of FSH compared to the preimplantation FSH levels was observed. This decrease in FSH levels is due to the implants’ ability to produce estrogen thereby restoring the HPG axis in the ovariectomized mice. We used ovaries from 6–8 days old BALB/c mice due to the presence of a higher proportion of primordial/primary follicles, which could withstand the initial hypoxic environment post-transplantation. Following transplantation, a restoration of estrous activity could be due to the activation of the implanted follicles and recruitment into the growing pool with every estrous cycle. Growing follicles were consistently present in the histological analysis of the encapsulated ovarian implants in mice showing decreased FSH levels.

Sittadjody *et al*.^49,50^ reported that multilayered constructs prepared with cells isolated from ovarian tissues and encapsulated in alginate secreted sex hormones *in vitro* and restored HPG axis in syngeneic studies in rats. Importantly, this study demonstrated superior short- and long-term outcomes from delivery of ovarian cells compared to pharmacological HRT (pHRT) in ovariectomized rats, which may have important clinical implications. In the current study we investigated whether the allogeneic ovarian tissue was immune-protected by measuring allo-specific antibodies and T-cell infiltration of grafts. In contrast to the non-encapsulated or failed ovarian allografts that presented with elevated levels of allo-specific IgG and CD8+ lymphocyte graft infiltration, allo-specific IgG in the sera of mice receiving the dual encapsulated ovarian tissue were undetectable and there was no lymphocyte infiltration of the implants. Mice with elevated allo-specific IgG and CD8 T cells corresponded with the failure to restore HPG axis. These mice had elevated FSH levels indicating that the implanted allogeneic ovarian tissue was rejected by the host immune system.

In summary, the approach of hydrogel-based immunoisolation presents a minimally invasive and a robust way to restore hormonal balance in mice. The basic biology and the factors regulating folliculogenesis are similar in mouse and humans, however further comprehensive and mechanistic studies in large animals will establish the importance of vascularization and the advantages over pharmacological regimens currently available. Because, this cell-based therapy delivers hormones in a pulsatile self-regulating manner, adverse side effects observed with pharmacological treatments can be avoided. This report is the first to demonstrate the feasibility and application of a tunable PEG-based hydrogel to encapsulate allogeneic ovarian tissue and to restore endocrine function in ovariectomized mice preventing rejection. Future non-human primate studies will be conducted to assess the ability of the Dual PEG capsule to support corpus luteum formation; capsule modification may be required to promote vasculature formation and greater diffusion of necessary metabolites and nutrients without the risk of immune rejection.

## Supplementary information

Supplemental Figure 1

## Data Availability

The datasets generated during and/or analyzed during the current study are available from the corresponding author on reasonable request.
