# Supplementary material for: Encapsulation of ovarian allograft precludes immune rejection and promotes restoration of endocrine function in immune-competent ovariectomized mice
Source: Sci Rep. 2019 Nov 12;9:16614. doi: 10.1038/s41598-019-53075-8 (PMC6851353; doi:10.1038/s41598-019-53075-8)
Supplement: Supplementary file 1 — Supplemental Figure 1 [file 41598_2019_53075_MOESM1_ESM.pdf]

Encapsulation of ovarian allograft precludes immune rejection and promotes restoration of endocrine function in immune-competent ovariectomized mice

James Ronald Day*[,1], Anu David*[, 1], Mayara Garcia de Mattos Barbosa[4,5], Margaret Ann Hammersley[1], Marilia Cascalho[4,5], Ariella Shikanov[1,2,3]

[1]Department of Biomedical Engineering, University of Michigan, Ann Arbor, USA
[2]Department of Macromolecular Science & Engineering, University of Michigan, Ann Arbor, USA
[3]Department of Obstetrics and Gynecology, University of Michigan, Ann Arbor, USA
[4]Department of Surgery, University of Michigan, Ann Arbor, USA
[5]Department of Microbiology & Immunology, University of Michigan, Ann Arbor, USA

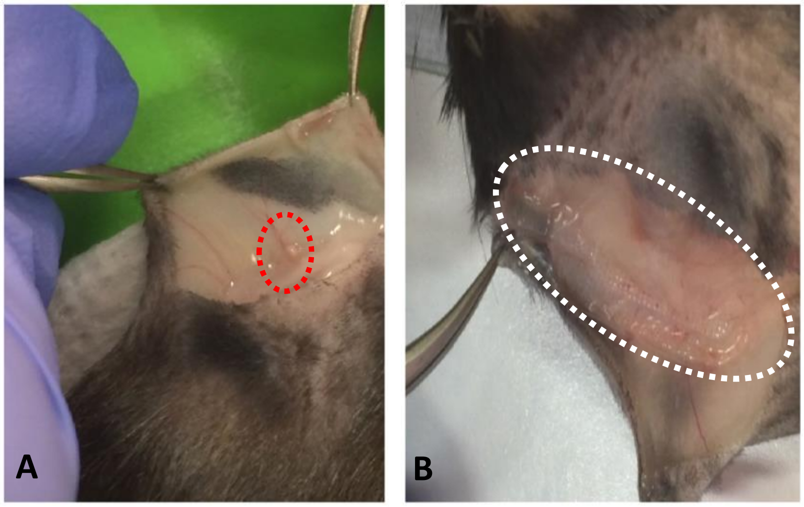

Supplemental Figure 1: **(A)** Non-encapsulated allogeneic ovarian tissue and (B) TheraCyte after 60 days of subcutaneous implantation in ovariectomized mice
